# Supplementary material for: Evaluating Large Language Models in extracting cognitive exam dates and scores
Source: PLOS Digit Health. 2024 Dec 11;3(12):e0000685. doi: 10.1371/journal.pdig.0000685 (PMC11634005; doi:10.1371/journal.pdig.0000685)
Supplement: S1 Section — (DOCX) [file pdig.0000685.s002.docx]

**S1 Section. Characteristics of Patients with Cognitive Tests With and Without MRI in the System, vs the subset of those with an MRI in the system.**

| **Feature** | **Patients with cognitive score and with or without MRI in the system N=52949** | **Patients with cognitive score and an MRI, selected for the study N=458** |
| --- | --- | --- |
| **Patient demographics** | | |
| Age at time of note (mean(sd)) | 69.64 (12.5) | 73.68 (14.01) |
| Gender | | |
| Female (%) | 47.84 % | 52.54 % |
| Male(%) | 38.77 % | 47.46 % |
| Unknown or X | 13.39 % | 0 |
| Race | | |
| Asian | 4.86 % | 5.90 % |
| Black | 9.85 % | 8.52 % |
| White | 55.16 % | 72.93 % |
| American Indian | 0.77 % | 0.22 % |
| Unknown | 29.36 % | 12.45 % |
