# Supplementary material for: Evaluating Large Language Models in extracting cognitive exam dates and scores
Source: PLOS Digit Health. 2024 Dec 11;3(12):e0000685. doi: 10.1371/journal.pdig.0000685 (PMC11634005; doi:10.1371/journal.pdig.0000685)
Supplement: S2 Section — All note shifts for one patients are consistent). (DOCX) [file pdig.0000685.s003.docx]

**Section S2. Examples of the notes and corresponding output that is produced by ChatGPT. (Dates for each patient note and ChatGPT responses are shifted by a random year and month, to preserve anonymous nature of notes. All note shifts for one patients are consistent.)**

| **Note Example 1:**  Mar 22, 2023: Patient was last seen in 4/2022. Since last visit, she had brain MRI which showed no acute intracranial pathology. Few SVID. Her EEG is normal. She has not done neuropsych test yet due to insurance coverage.   Today, she reports her headache and dizziness improved since last visit. Her short term memory impairment is still active, affects her daily activities. No disorientation, confusion or hallucination.    History:  Patient had Covid-19 infection in 5/2021. She  has frequent headache , dizziness, brain foggy since then. She forgot thing happened minutes ago and repeated same questions frequently. She has word finding difficulty during conversation. She has short attention span.   MMSE was 28/30 in last visit. |
| --- |
| **ChatGPT response:**  {  "MMSE": [     {      "score": "28/30",             "date": "4/2022"    }     ],    "CDR": []  } |

| **Note Example 2:**  January 2016: [……] Cognitive testing was repeated:         Pre CLD Post CLD     DATE  10/09/16  09/05/16  05/10/17  09/04/17  09/07/17  08/09/17  11/30/17    GDS  2  2  2  2  2   2    MMS  30  30  29  30    30    MOCA  30  29  26     30               Written Arithmetic  1       1    Number Writing  1       1    Spelling  1       1    Clock  1       1               DSST    53  56  58  58  51    PSPC  91   58  55  56  58  64               Trail Making A             trial 1 31   32.9  35.6  32.6  29.6  38.3     trial 2    36  25.1  30.7      trial 3    27.8  21.9  30.3      trial 4    29.6  27.7       mean 31   32.9  32.3  26.8  30.2  38.3     errors   0   1  0  1               Trail Making B            trial 1 55.0   84.3  58.7  53.4  62.7  59.7     trial 2    60.2  76.2  46.7      trial 3    57  61.2  56.2      trial 4    39.9  52.5  73.3      mean 55.0   84.3  54.0  60.8  59.7  59.7     errors   1  1  3  3  0        118/78@78 bpm, RR = 18 |
| --- |
| **ChatGPT Response:**   ChatGPT Response:   "MMSE": [      {        "score": 30,        "date": "January 2016"      },      {        "score": 30,        "date": "09/05/17"      },      {      "score": 29,        "date": "05/10/18"      },      {        "score": 30,        "date": "09/04/18"      },      {        "score": 30,        "date": "09/07/18"      },  {        "score": 30,        "date": "11/30/18"   }    ],    "CDR": []} |

| **Note Example 3:**  Jan 6 2017: [……] Last visit was 10/14/16. MMSE was 29/30. [……] Coordination: finger-nose intact; heel-shin intact; rapid alternating finger taps, hand opening/closing,foot taps, leg taps are mildly slow but regular Gait: rises without using hands, normal stance, normal stride, normal rate, normal arm swing, no retropulsion, able to tandem Sensory: intact light touch Reflexes: 2+ bi, 2+ br, 2+ tri, 2+ patellar, 1+ Achilles, plantar response flexor, +palmomental, negative grasp, glabellar, snout MMSE 29/30 CDR 0.5 Assessment: He has amnestic MCI due to Alzheimer disease. [……] |
| --- |
| **ChatGPT Response:**   ChatGPT Response:  {  "MMSE": [  {  "score": "29/30",  "date": "10/14/16"  },  {  "score": "29/30",  "date": "Jan 06, 2017"  }  ],  "CDR": [  {  "score": "0.5",  "date": "Jan 06, 2017"  }  ]} |

| **Note Example 4:**  June 26 2018: [……] October 2017 with increasing concerns regarding distractibility, cognitive slowing and inefficiency. Wife believes an "ADD" -type learning style has always been present, but these days, the distractibility is interfering with some of his daily activities. [……] He endorsed no significant depression symptoms. Elemental neurological exam notable for slightly increased tone, a sway with Romberg, brisk DTRs, and and unsteady tandem gait. Overall, patient fits a Clinical Dementia Rating of 0.5, or, "questionable" consistent with mild cognitive impairment. The pattern of deficits is somewhat unusual with disproportionate visuospatial processing/ attention/ executive dysfunction >> memory. [……] Staging now between CDR 0.5 and 1.0 (MCI and mild dementia). [……] |
| --- |
| **ChatGPT Response:**   ChatGPT Response:  {  "MMSE": [  {  "score": "22/30",  "date": "June 26, 2018"  }  ],  "CDR": [  {  "score": "0.5",  "date": "October 2017"  },  {  "score": "between 0.5 and 1.0",  "date": "June 26, 2018"  }  ]} |
