# Supplementary material for: Evaluating Large Language Models in extracting cognitive exam dates and scores
Source: PLOS Digit Health. 2024 Dec 11;3(12):e0000685. doi: 10.1371/journal.pdig.0000685 (PMC11634005; doi:10.1371/journal.pdig.0000685)
Supplement: S3 Section — (DOCX) [file pdig.0000685.s004.docx]

**Section S3. Parsing the JSON results**

ChatGPT and LlaMA-2 were instructed to provide answers in JSON format. The human expert reviewers were instructed to provide ground truth data also in JSON format. To facilitate the review process for expert reviewers and reduce errors due to word choices, human reviewers were recommended to start from ChatGPT’s answer and correct the incorrect parts. Both results were then parsed automatically and turned into a list of (score, date) tuples, (four lists of tuples per note: 1) ChatGPT’s MMSE, 2) ChatGPT’s CDR, 3) reviewer’s MMSE, 4) reviewer’s CDR.) MMSE and CDR scores were analyzed individually. To normalize the JSON entries, any entry corresponding to the “score” which had obvious wrong test types (i.e “MoCA”, “GDS”) was automatically excluded.
