# Supplementary material for: Evaluating Large Language Models in extracting cognitive exam dates and scores
Source: PLOS Digit Health. 2024 Dec 11;3(12):e0000685. doi: 10.1371/journal.pdig.0000685 (PMC11634005; doi:10.1371/journal.pdig.0000685)
Supplement: S4 Section — (DOCX) [file pdig.0000685.s005.docx]

**Section S4. JSON responses**

Two (out of 722) responses from ChatGPT were not in correct JSON format and failed automatic parsing. These two cases were both reporting “no MMSE or CDR.” Another 26 of the remaining 722 ChatGPT JSON results had “MMSE_Scores” and “CDR_Scores” as the JSON entry, while the rest had “MMSE” and “CDR” as the entries. The JSON results of 79 notes had additional entries, primarily including other cognitive tests (47 “MoCA” and 20 “GDS” being the most frequent wrong tests). These additional entries in JSON results of both ChatGPT and human reviewers were automatically excluded from analysis.

All LlaMA2 responses were in correct JSON format and were analyzed in the exact same manner as the responses from ChatGPT.

Of all 1031 (309×2 + 413) human expert responses, 131 were not in correct JSON format and failed automatic parsing. Most, 92, of those were missing a single formatting character (e.g. comma, bracket) and were manually corrected. The remaining 39 were long entries not provided in JSON format that would have required substantial edits and were consequently excluded from analysis. As a result, 12 of the notes (3 coming from the double-reviewed notes) were excluded from further analysis. In the remaining 710 notes (306 of which were double-reviewed), all parsed ground truth and ChatGPT and LlaMA2 instances of MMSE and CDR tests were analyzed for computing accuracy, precision, and recall. ChatGPT’s and LlaMA-2’s (micro- and macro-) precision and recall for MMSE and CDR items are included in Table 4.
