# Supplementary material for: Evaluating Large Language Models in extracting cognitive exam dates and scores
Source: PLOS Digit Health. 2024 Dec 11;3(12):e0000685. doi: 10.1371/journal.pdig.0000685 (PMC11634005; doi:10.1371/journal.pdig.0000685)
Supplement: S5 Section — (DOCX) [file pdig.0000685.s006.docx]

**Section S5. Qualitative analysis of error instances of ChatGPT**

We analyzed 31 notes from the double-reviewed notes where ChatGPT’s predictions for CDR had any incorrect elements in them. In 3 notes, there was no sign of a CDR score and the result was hallucinated without an obvious reason. In 4 cases, the date was wrong but the score was correct. The remaining 24 notes’ errors were all due to GPT mistakenly reporting results of another test (e.g. GDS, CD4, and Cup/Disk Ratio, also abbreviated CDR) instead of CDR. We similarly analyzed the 52 notes which had any errors for the MMSE predicted list. These notes’ errors included 19 with an error only in the reported date while the score was correct; 17 cases of wrong test scores (i.e mostly MoCA, only 2 MiniCog and 1 MSK cases) being picked instead of MMSE; 4 cases of inconsistency in the note itself; 2 typos (one case where the note said “AOx3” for the score, another where MMSE was written as “MMS E”) according to the reviewer comments; 3 cases of hallucinations without specific reasons; 4 cases of non-numeric MMSE values (i.e. “deferred'' or “preserved”); 1 case of reporting partial MMSE scores instead of the total MMSE score; 1 case was missed from a table that listed 7 MMSE score/dates that were easy to see in the HTML version of the note but confusing in text-only version; and finally, 1 case was actually correct and the error was due to not considering “not found”' equal to an empty list.

For MMSE, we also reviewed the errors of LlaMA-2. Among the 98 double-reviewed notes with any error in the results according to LlaMA-2, we observed the following errors: 27 cases had total hallucination. In 19 cases other scores were reported instead of MMSE. There were 25 missed scores, and 23 cases where the wrong date was reported for the right score.
