# Supplementary material for: Evaluating Large Language Models in extracting cognitive exam dates and scores
Source: PLOS Digit Health. 2024 Dec 11;3(12):e0000685. doi: 10.1371/journal.pdig.0000685 (PMC11634005; doi:10.1371/journal.pdig.0000685)
Supplement: S1 Table — (DOCX) [file pdig.0000685.s007.docx]

**S1 Table. Diagnosis Criteria for Cognitively Normal, Mild Cognitive Impairment and Mild Alzheimer’s Disease Dementia in ADNI cohorts.**

| Criteria | ADNI |
| --- | --- |
| Cogniti-vely Normal | ● No Memory Complaints aside from those common to other normal subjects of that age range.  ● Normal memory function documented by scoring at specific cutoffs on the Logical Memory II subscale (delayed Paragraph Recall) from the Wechsler Memory Scale - Revised (the maximum score is 25): a) greater than or equal to 9 for 16 or more years of education b) greater than or equal to 5 for 8-15 years of education c) greater than or equal to 3 for 0-7 years of education.  ● Mini-Mental State Exam score between 24 and 30 (inclusive) (Exceptions may be made for subjects with less than 8 years of education at the discretion of the project director).  ● Clinical Dementia Rating = 0. Memory Box score must be 0.  ● Cognitively normal, based on an absence of significant impairment in cognitive functions or activities of daily living. |
| MCI | ● Memory complaint by subject or study partner that is verified by a study partner.  ● Abnormal memory function documented by scoring below the education adjusted cutoff on the Logical Memory II subscale (Delayed Paragraph Recall) from the Wechsler Memory Scale – Revised (the maximum score is 25): a) less than or equal to 8 for 16 or more years of education b) less than or equal to 4 for 8-15 years of education c) less than or equal to 2 for 0-7 years of education.  ● Mini-Mental State Exam score between 24 and 30 (inclusive) (Exceptions may be made for subjects with less than 8 years of education at the discretion of the project director).  ● Clinical Dementia Rating = 0.5. Memory Box score must be at least 0.5.  ● General cognition and functional performance sufficiently preserved such that a diagnosis of Alzheimer’s disease cannot be made by the site physician at the time of the screening visit. |
| AD | ● Memory complaint by subject or study partner that is verified by a study partner.  ● Abnormal memory function documented by scoring below the education adjusted cutoff on the Logical Memory II subscale (Delayed Paragraph Recall) from the Wechsler Memory Scale – Revised (the maximum score is 25): a) less than or equal to 8 for 16 or more years of education b) less than or equal to 4 for 8-15 years of education c) less than or equal to 2 for 0-7 years of education.  ● MMSE between 20 and 26 (inclusive) (Exceptions may be made for subjects with less than 8 years of education at the discretion of the protocol PI).  ● Clinical Dementia Rating = 0.5, 1.0 ➪ NINCDS/ADRDA criteria for probable AD. |
