# Supplementary material for: Evaluating Large Language Models in extracting cognitive exam dates and scores
Source: PLOS Digit Health. 2024 Dec 11;3(12):e0000685. doi: 10.1371/journal.pdig.0000685 (PMC11634005; doi:10.1371/journal.pdig.0000685)
Supplement: S2 Table — (DOCX) [file pdig.0000685.s008.docx]

**S2 Table. Description of the errors encountered by ChatGPT API**

| Error | Meaning |
| --- | --- |
| Azure content management violation | The content of the note activated the “content filtration” classifier that monitors content sent to the ChatGPT API for harmful content or content that violates Asure’s usage policy.  No additional information is made available to us about what aspect of the clinical note triggered this classifier.  Azure OpenAI Service includes a content filtering system that works alongside core models. This system works by running both the prompt and completion through an ensemble of classification models aimed at detecting and preventing the output of harmful content. The content filtering system detects and takes action on specific categories of potentially harmful content in both input prompts and output completions. Variations in API configurations and application design may affect completions and thus filtering behavior. |
| API timeouts | When making requests to the OpenAI API, there is a predefined time period within which the API expects to provide a response. If the API fails to respond within that time frame, it generates a "Request timed out" error.  This error may be due to reaching limits of number of requests per second, or other factors. |
| Maximum length limit error | ChatGPT 8K allows 8000 tokens in the input. This error occurs when then input includes more than 8K tokens in the input. |
