# Supplementary material for: Evaluating Large Language Models in extracting cognitive exam dates and scores
Source: PLOS Digit Health. 2024 Dec 11;3(12):e0000685. doi: 10.1371/journal.pdig.0000685 (PMC11634005; doi:10.1371/journal.pdig.0000685)
Supplement: S3 Table — (DOCX) [file pdig.0000685.s009.docx]

**S3 Table. The prompt (and the full request JSON for the task) for ChatGPT. CLINICAL_NOTE would include the date of the note (from EPIC) + “:” + the text-only content of the notes.**

| openai.api_type = "azure"  openai.api_version = "2023-03-15-preview"  response = openai.ChatCompletion.create(  engine="GPT4",  messages = [{"role":"system",  "**content**":"You are an AI assistant that helps doctors find information from recorded clinical notes that talk about the cognitive health of patients. \n\nPlease identify and extract all instances of Mini Mental Status Exam (MMSE) and Cognitive Dementia Rating (CDR) scores mentioned in the given text, along with the dates that you think the tests were administered, and provide the information in JSON format."}, {"**role**":"user","**content**":CLINICAL_NOTE}],  temperature=0,  max_tokens=800,  top_p=0.95,  frequency_penalty=0,  presence_penalty=0,  stop=None) |
| --- |
