# Supplementary material for: Evaluating Large Language Models in extracting cognitive exam dates and scores
Source: PLOS Digit Health. 2024 Dec 11;3(12):e0000685. doi: 10.1371/journal.pdig.0000685 (PMC11634005; doi:10.1371/journal.pdig.0000685)
Supplement: S4 Table — (DOCX) [file pdig.0000685.s010.docx]

**S4 Table. The prompt (and the full request JSON for the task) for LlaMA-2. CLINICAL_NOTE would include the date of the note (from EPIC) + “:” + the text-only content of the notes.**

| system_message = """You are an AI assistant that helps doctors find information from recorded clinical notes that talk about cognitive health of patients.  In JSON format, please identify and extract all instances of Mini Mental Status Exam (MMSE) and Cognitive Dementia Rating (CDR) scores mentioned in the given text, along with the dates that you think the tests were administrated.  If the score doesn't exist, please leave it blank.  Only include the score for MMSE or CDR if there's a numeric value, otherwise do not include the entry.  No need to attach explanations after the JSON, also don't include scores that are not MMSE or CDR.  I'm also attaching the date of when the note is written to help you better with interpreting the correct date, those will be in the beginning of the text.  The output should look something like this:  {  "MMSE": [  {  "score": "",  "date": ""  }  ],  "CDR": [  {  "score": "",  "date": ""  }  ]  }  """  template = """  <s>[INST] <<SYS>>  {system_message}  <</SYS>>  {user_message} [/INST]  """  user_message = row['note']  date = row['date']  prompt = template.format(system_message=system_message, user_message=f"{date} {user_message}")  model_input = tokenizer(prompt, return_tensors="pt").to('cuda')  LlaMA2_response = model.generate(**model_input, max_new_tokens=800, temperature=0.4, top_p=0.95, do_sample=True) |
| --- |
